# Reference gene selection for quantitative reverse transcription-polymerase chain reaction normalization during *in vitro *adventitious rooting in *Eucalyptus globulus *Labill

**DOI:** 10.1186/1471-2199-11-73

**Published:** 2010-09-20

**Authors:** Márcia R de Almeida, Carolina M Ruedell, Felipe K Ricachenevsky, Raul A Sperotto, Giancarlo Pasquali, Arthur G Fett-Neto

**Affiliations:** 1 Center for Biotechnology, Federal University of Rio Grande do Sul, P.O. Box 15005, 91501-970 Porto Alegre, RS, Brazil; 2 Department of Botany, Federal University of Rio Grande do Sul, P.O. Box 15005, 91501-970 Porto Alegre, RS, Brazil

## Abstract

**Background:**

*Eucalyptus globulus *and its hybrids are very important for the cellulose and paper industry mainly due to their low lignin content and frost resistance. However, rooting of cuttings of this species is recalcitrant and exogenous auxin application is often necessary for good root development. To date one of the most accurate methods available for gene expression analysis is quantitative reverse transcription-polymerase chain reaction (qPCR); however, reliable use of this technique requires reference genes for normalization. There is no single reference gene that can be regarded as universal for all experiments and biological materials. Thus, the identification of reliable reference genes must be done for every species and experimental approach. The present study aimed at identifying suitable control genes for normalization of gene expression associated with adventitious rooting in *E. globulus *microcuttings.

**Results:**

By the use of two distinct algorithms, *geNorm *and *NormFinder*, we have assessed gene expression stability of eleven candidate reference genes in *E. globulus*: *18S, ACT2, EF2, EUC12, H2B, IDH, SAND, TIP41, TUA, UBI *and *33380*. The candidate reference genes were evaluated in microccuttings rooted *in vitro*, in presence or absence of auxin, along six time-points spanning the process of adventitious rooting. Overall, the stability profiles of these genes determined with each one of the algorithms were very similar. Slight differences were observed in the most stable pair of genes indicated by each program: *IDH *and *SAND *for *geNorm*, and *H2B *and *TUA *for *NormFinder*. Both programs indentified *UBI *and *18S *as the most variable genes. To validate these results and select the most suitable reference genes, the expression profile of the *ARGONAUTE1 *gene was evaluated in relation to the most stable candidate genes indicated by each algorithm.

**Conclusion:**

Our study showed that expression stability varied between putative reference genes tested in *E. globulus*. Based on the *AGO1 *relative expression profile obtained using the genes suggested by the algorithms, *H2B *and *TUA *were considered as the most suitable reference genes for expression studies in *E. globulus *adventitious rooting. *UBI *and *18S *were unsuitable for use as controls in qPCR related to this process. These findings will enable more accurate and reliable normalization of qPCR results for gene expression studies in this economically important woody plant, particularly related to rooting and clonal propagation.

## Background

*Eucalyptus *sp. is one of the most planted hardwood genus in the world [1], mainly because of its applications for generating wood and paper products. Brazil is the largest world producer of eucalypts for short fiber pulp [2], thanks to clonal forests developed from elite material with high productivity [3]. In southern Brazil and temperate areas, especially Mediterranean Europe, Portugal and Chile, *Eucalyptus globulus *and its hybrids are of interest for the cellulose industry due to their relatively high frost resistance and low lignin content, which facilitates cellulose extraction [4]. On the other hand, *E. globulus *is generally considered recalcitrant to rooting [5,6]. The commercial eucalypt forests are generally formed through vegetative propagation which has adventitious rooting as a key step [7]. This developmental process can be divided in two main steps, each with its own requirements and characteristics: (1) induction step, which involves biochemical and molecular events, without visible morphological changes; and (2) formation step, which consists of cellular divisions involved in both root meristem organization and primordium establishment, followed by root elongation and emergence out of the cutting [8].

Multiple factors can interfere with adventitious rooting, such as phytohormones, phenolic compounds, nutritional conditions and genetic characteristics [9]. Among phytohormones, auxins play a central role in rooting capacity [10], particularly its endogenous content and transport rate [11]. However, concentrations that are beneficial to root induction can block its elongation [7]. The shoot apex is the main source of endogenous auxin. Stems have a specific basipetal active transport through vascular parenchyma carried out by both influx (AUX1) and efflux (PIN) carriers [12,13].

*Eucalyptus globulus *needs exogenous auxin application for an adequate root development *in vitro *[8], but it is unknown if this occurs because of defficiencies in auxin content, transport or perception mechanisms, or due to some other non-auxin related reason that can block spontaneous rooting responses. At gene expression level, little is known about the formation of root meristems in woody plants and even less concerning the effect of auxins on this process [14].

To evaluate changes in gene expression, reverse-transcription followed by quantitative, real-time polymerase chain reaction (qPCR) is one of the most widely used methods. Some of the advantages of qPCR are high sensitivity and specificity, speed, ease of use and capacity to carry out simultaneous measurements of gene expression in several different samples for a limited number of genes [15-17]. To accurately and reproducibly quantify gene expression, some factors should be taken into account, such as quality and amount of starting material, presence of inhibitors in different samples, primer design, RNA extraction and reverse-transcription efficiencies [16]. Therefore, selection of an appropriate normalization strategy is essential for obtaining an accurate and reliable quantification of gene expression levels [15,16,18]. The most commonly applied approach for normalization of qPCR is the use of one or more endogenous reference genes [18-20], which will undergo the same type of variation and preparation steps throughout the assay as the target genes.

An ideal reference gene, also known as housekeeping or internal control gene, is assumed to be constitutive, i.e., it should be expressed at a constant level in the majority of the study organism cells, through different developmental stages and in different organs. Besides, its expression is assumed to be unaffected by experimental parameters [21]. Genes involved in basic cellular processes, such as cell structure maintenance or primary metabolism, are often chosen as normalizers [22]. However, the transcript levels of these putative reference genes are not always stable and their systematic use without previous validation may lead to the misinterpretation of results. Indeed, the previous assurance of at least one stable gene expression in all variable situations tested is recommended to accurately validate the expression of genes under analysis [22-27]. In recent years, a large number of reference gene validation attempts have been reported for plants, but most of them have covered model and crop species: rice [28,29], sugarcane [30], *Arabidopsis thaliana *[22,31], potato [26], grape [32], barley [33], *Brachypodium *sp. [34], soybean [35,36], tomato [37], *Brachiaria *sp. [38], coffee [39], peach [40], wheat [41], chicory [42], cotton [43], cucumber [44], longan tree [45], *Petunia *sp. [46], ryegrass [47], rapeseed [48] and tobacco [49]. Just a few studies have focused on forest genera, such as poplar [25,27] and *Eucalyptus *sp. [50]; however, there is a lack of investigations covering *in vitro *growth or adventitious rooting.

**Figure 1:**
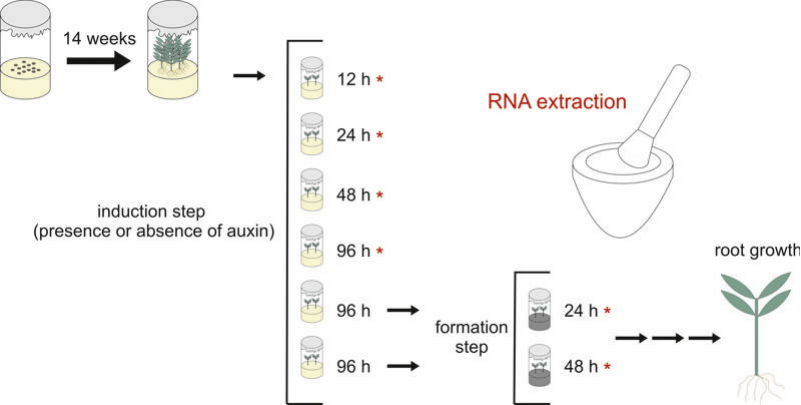
**Stages of *in vitro *adventitious rooting in *Eucalyptus globulus***. After seeding, seedlings remained 14 weeks in germination medium. After this time, apical microcuttings were obtained and used in adventitious rooting experiments. The microcuttings were kept in induction medium (presence or absence of 10 mg l^-1 ^IBA) for 12, 24, 48 or 96 h, depending on the harvest time. For the harvest points in formation medium, the microcuttings remained 96 h in induction medium and after that were transferred to formation medium (devoid of auxin and with activated charcoal). In this case, the samples were collected after 24 and 48 h. The samples harvested at 12, 24, 48 and 96 h of induction step and at 24 and 48 h of formation step were submitted to RNA extraction and used for the analyses.

The aim of this study was to evaluate the stability of 11 putative reference genes for the purpose of normalization in studying *E. globulus *gene expression during *in vitro *adventitious rooting (Figure 1). Statistical methods implemented in *geNorm *[24] and *NormFinder *[51] were used and compared in this evaluation. In addition, to further validate the reference genes, an expression analysis of a putative *E. globulus *orthologue of the *A. thaliana ARGONAUTE1 (AGO1) *gene during rooting of microcuttings was carried out.

## Results

To identify the most stable genes in *E. globulus *microcuttings rooted *in vitro*, we selected 11 candidate reference genes to validate by qPCR. The gene expression stability of the candidates was evaluated in a set of 12 samples covering six different stages along adventitious rooting time in the presence or absence of the phytohormone auxin. The genes included: 18S ribosomal RNA (*18S*) [25-27,40], Actin 2/7 (*ACT2*) [24,40], Translation elongation factor 2 (*EF2*) [40], Histone H2B (*H2B*) [52], NADP Isocitrate Dehydrogenase (*IDH*) [53-55], Polyubiquitin (*UBI*) [25,27], SAND protein (*SAND*) [22,31], TIP41-like protein (*TIP41*) [22,27,31,37], Alpha-tubulin (*TUA*) [25], an *Eucalyptus *ortholog of *Arabidopsis thaliana *expressed protein without determined function (*33380*) [22,27,31], and a putative RNA binding protein (*EUC12*) previously shown to be constitutive in different *Eucalyptus *organs (unpublished results). The characteristics of each of the listed genes, such as gene name, *A. thaliana *ortholog locus, *A. thaliana *locus description, *GenBank *accession number, function and *E-value *are provided in Table 1. All *Arabidopsis *genes were used simply to identify the corresponding orthologs in *Eucalyptus *using the *Genolyptus project *data base. The specificity of the amplifications was confirmed by the presence of a single band of expected size for each primer pair in agarose gel electrophoresis after PCRs employing either cDNA or chromosomal DNA as templates, and by the observation of single-peak melting curves of the qPCR products (data not shown). No primer dimers or other products resulted from non-specific amplification. Amplification efficiencies of PCRs ranged from 1.790 for *EF2 *to 1.972 for *H2B *(Table 2).

**Table 1:** Description of reference genes used for qPCR in *Eucalyptus globulus.*

Gene symbol	Gene name	*Arabidopsis thaliana*'s best hit locus	*Arabidopsis thaliana *locus description	Putative *E. globulus *orthologs GeneBank accession number	Function	E-value (tblastx)
*18S*	RNA ribosomal 18S	At3g41768	RNA ribosomal 18S	HO048251	Cytosolic small ribosomal subunit, translation	0,0

*ACT2*	Actin 2/7	At5g09810	Actin 2/7	HO048249	Structural constituent of cytoskeleton	0,0

*EF2*	Translation elongation factor 2	At1g56070	Translation elongation factor 2, putative	HO048253	Translation factor activity, nucleic acid binding	e-176

*EUC12*	Putative RNA bindig protein	*	*	HO048248	Unknown	e-78

*H2B*	Histone H2B	At5g59910	Histone H2B, putative	HO048243	Strutural constituent of the eukaryotic nucleosome core	e-66

*IDH*	NADP-Isocitrate dehydrogenase	**	**	HO048252	Carbohydrate metabolism	**

*UBI*	Polyubiquitin	At4g05050	Polyubiquitin gene, belongs to a subtype group with UBQ10 and UBQ14	HO048245	Signalling complexes for protein degradation, translation control, DNA repair, endocytosis regulation, protein traffic	e-144

*SAND*	SAND protein	At2g28390	SAND family protein	HO048246	Intracellular vesicular transport, biogenesis and vacuole signalling	e-68

*TIP41*	TIP41-like protein	At4g34270	TIP41-like family protein	HO048247	Unknown	e-78

*TUA*	Alpha-tubulin	At5g19780	Alpha tubulin-5	HO048250	Structural constituent of cytoskeleton, microtubule-based processes	e-134

*33380*	Expressed protein	At4g33380	Expressed sequence	HO048244	Unknown	e-74

* Gene assumed as constitutive in microarray studies employing leaf and vascular tissues of fully grown *E. globulus *and *E. grandis *trees (unpublished results). ** Gene obtained from Carvalho *et al. *(2008) [55].

**Table 2:** Primer sequences and amplicon characteristics for each of the 11 reference genes and for the *Argonaute 1 *gene of interest.

Gene symbol	Primer sequence (5' → 3') Forward/Reverse	Amplicon length (bp)	Annealing temperatures (°C)	Amplification efficiency
*18S*	TGACGGAGAATTAGGGTTCG/ CCGTGTCAGGATTGGGTAAT	100	60	1.860

*ACT2*	TCCACCATGTTCCCTGGTAT/ ACCTCCCAATCCAGACACTG	124	60	1.935

*EF2*	GCGTTCCCTCAGTGTGTCTT/ GGTCATCTGCTCCTTCAAGC	126	60	1.790

*EUC12*	GCGTGGTTCTTGGATCACTA/ TGGTGACAAAGTCAGGTGCT	114	59	1.807

*H2B*	GAAGAAGCGGGTGAAGAAGA/ GGCGAGTTTCTCGAAGATGT	145	60	1.972

IDH *	CTGTTGAGTCTGGAAGATGAC/ CATTTAATTCCTCCCCAACAAA	271	60	1.892

*UBI*	AGAAGGAATCGACCCTCCAC/ CCTTGACGTTGTCAATGGTG	126	60	1.888

*SAND*	CCATTCAACACTCTCCGACA/ TGTGTGACCCAGCAGAGTAAT	143	59	1.919

*TIP41*	GAACAAAAGCTCGGGACATC/ CAACCAGCAAGAGCATCAAA	122	60	1.938

*TUA*	ACCGGTTGATCTCTCAGGTG/ TAAGGGACCAGGTTGGTCTG	103	60	1.845

*33380*	TCCAGAGTGCAATGCTGAAC/ CCCCTCGTCTGGCATACTTA	134	60	1.935

*AGO1*	TCTTGGGCTCGTTTCTCAGT GGAATTCGCCTAGACAGTGC	144	60	1.887

* Primer obtained from Carvalho *et al. *(2008) [55].

### Expression levels of reference gene candidates

**Figure 2:**
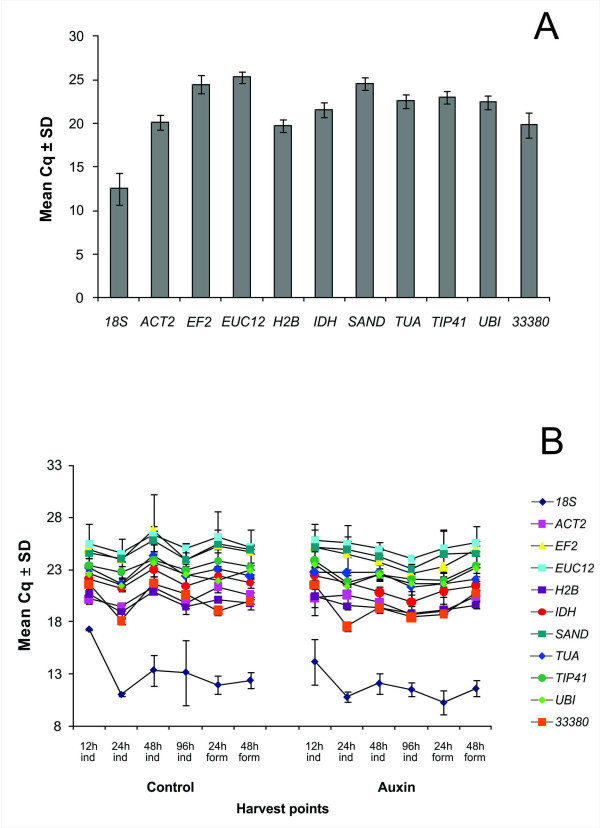
**RNA transcription level of reference genes tested, presented as Cq mean value ± Standard Deviation (SD)**. (A) Mean Cq of each reference gene. (B) Transcription level profile of reference genes along the adventitious rooting process. ind: induction step; form: formation step. Auxin: indicates addition of 10 mg l^-1 ^Indol Butyric Acid (IBA) in induction step culture medium; control: indicates absence of IBA in induction step culture medium.

Not surprisingly, some variations on Cq values, i.e. number of cycles needed for the amplification-related fluorescence to reach a specific threshold level of detection, occurred among the reference genes tested. Transcripts of *18S *were the most abundant (mean Cq = 12.48 ± 1.87), followed by *H2B, 33380, ACT2, IDH, UBI, TUA, TIP41, EF2 *and *SAND*, whereas *EUC12 *was the least abundant (mean Cq = 25.30 ± 0.63) (Figure 2A). The expression profiles of all candidate reference genes along the adventitious rooting process are depicted in Figure 2B.

### Expression stability of reference gene candidates

Two programs were used to evaluate the stability of the 11 candidate reference genes: *geNorm *[24] and *NormFinder *[52]. Cq data were collected for all samples and transformed to relative quantities using the delta-Cq method developed by Livak and Schmittgen [56] (see Material and Methods section for details).

### *geNorm *analysis

**Figure 3:**
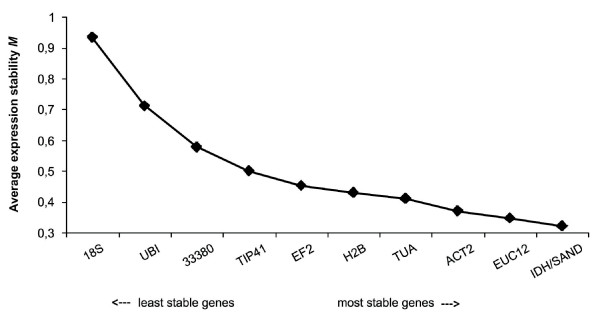
**Gene expression stability (*M*) and ranking of the 11 reference genes as calculated by *geNorm *in *Eucalyptus globulus *microcuttings during *in vitro *adventitious rooting**. The microcuttings were rooted in presence or absence of 10 mg l^-1 ^Indol Butyric Acid (IBA) in the induction step. A lower average expression stability *M *value indicates more stable expression.

**Figure 4:**
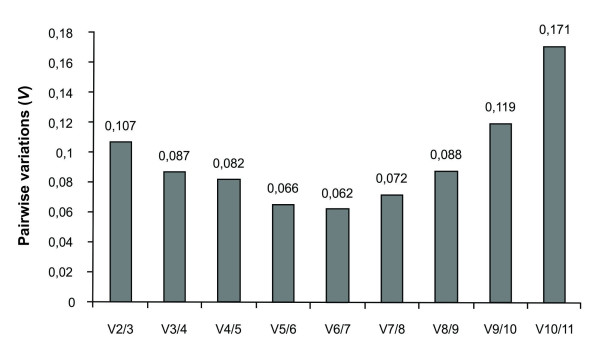
**Determination of the optimal reference gene number as calculated by *geNorm *for accurate normalization during *Eucalyptus globulus in vitro *adventitious rooting**. *geNorm *pairwise variation values (V values) are calculated by an algorithm which measures pairwise variation (Vn/n + 1) between two sequential normalization factors NFn and NFn + 1, where n is the number of genes involved in the normalization factor.

The average expression stability (*M *value) of all genes was calculated by *geNorm *(version 3.5). The *M *value is defined as the average pairwise variation of a particular gene with all other potential reference genes. The average *M *values of the candidate reference genes tested are shown in Figure 3 and summarized in Table 3. The *geNorm *program recommends using an *M *value below the threshold of 1.5 to identify reference genes with stable expression, but some authors propose the maximum value of 0.5 to obtain more accurate results [57,27]. Our results indicate *IDH *and *SAND *as the most stably expressed genes (i. e., with the lowest *M *value = 0.320) and *18S *as the least (*M *value = 0.935). To evaluate the optimal number of reference genes for reliable normalization, *geNorm *calculates the pairwise variation V_*n*_/V_*n*+1 _between the sequential ranked normalization factors NF_*n *_and NF_*n*+1 _to determine the effect of adding the next reference gene in normalization. The normalization factor is calculated based on the geometric average among the three most stable genes relative quantities and stepwise inclusion of the other genes in the order of their expression stability. A large pairwise variation implies that the added reference gene has a significant effect on normalization and should be included for calculation of a reliable normalization factor [24]. Considering the *cut-off *value of 0.15 proposed by Vandesompele *et al*. [24], below which the inclusion of an additional reference gene is not necessary, the use of the two most stably expressed genes (*IDH *and *SAND*) is sufficient for accurate normalization (V_2/3 _= 0.107) in *E. globulus in vitro *rooting (Figure 4). If needed, the *EUC12 *gene can be added to the analysis, resulting in a pairwise variation value of 0.087 (V_3/4 _= 0.087) (Figure 4).

**Table 3:** Ranking of candidate reference genes in decreasing order of expression stability calculated by *geNorm *and *NormFinder.*

Ranking order	*geNorm *(*M *value)	*NormFinder *(Stability value ± EB*)
1	*IDH/SAND (0.320)*	*H2B (0.031 *± 0.006*)*

2		*TUA (0.084 *± 0.067*)*

3	*EUC12 (0.346)*	*IDH (0.116 *± 0.082*)*

4	*ACT2 (0.370)*	*EUC12 (0.121 *± 0.087*)*

5	*TUA (0.410)*	*TIP41 (0.123 *± 0.091*)*

6	*H2B (0.429)*	*SAND (0.143 *± 0.124*)*

7	*EF2 (0.452)*	*33380 (0.161 *± 0.156*)*

8	*TIP41 (0.500)*	*EF2 (0.174 *± 0.183*)*

9	*33380 (0.579)*	*ACT2 (0.186 *± 0.215*)*

10	*UBI (0.712)*	*UBI (0.306 *± 0.566*)*

11	*18S (0.935)*	*18S (0.547 *± 1.833*)*

* EB = Error Bars

### *NormFinder *analysis

In addition to the analysis by the *geNorm *software, the expression stability of the candidate reference genes on the experimental samples was also evaluated by *NormFinder*. This program takes into account the intra- and inter-group variations for normalization factor calculation and the results are not affected by occasional co-regulated genes. The best candidate will be the one with the inter-group variation as close to zero as possible, and, at the same time, having the smallest errors bars possible [51]. A change in the indication of most stable genes was observed after *NormFinder *analysis when compared to *geNorm*, with *H2B *showing the most stable expression (near zero variation value and small error bars), followed by *TUA*. These two genes were also among the most stably expressed genes based on *geNorm *analysis, displaying *M *values below 0.5 (Figure 3). *UBI *and *18S *were the least stable genes (both with variation values farther from zero and larger error bars) (Table 3). When defining the best combination of two genes using *NormFinder*, once again both *H2B *and *TUA *were the genes of choice, with a stability value of 0.045 when used together (data not show).

### Relative expression profile of *AGO1*

To further validate the control genes and for determining the best choice of genes to use, the relative expression of a putative ortholog of *A. thaliana ARGONAUTE1 *(*AGO1) *gene in *E. globulus *was investigated during rooting. *AGO1 *encodes a micro RNA binding protein [58] involved in regulating the expression of *ARF17 *(*Auxin Response Factor 17*), an auxin response transcription factor with inhibitory action, possibly controling genes related to auxin homeostasis and adventitious root development in *A. thalina *[10]. The two gene pairs indicated by *geNorm *and *Normfinder *were used as references to evaluate *AGO1 *relative expression in eight samples, covering time points of adventitious root induction and formation, both under presence and absence of auxin during the induction step.

**Figure 5:**
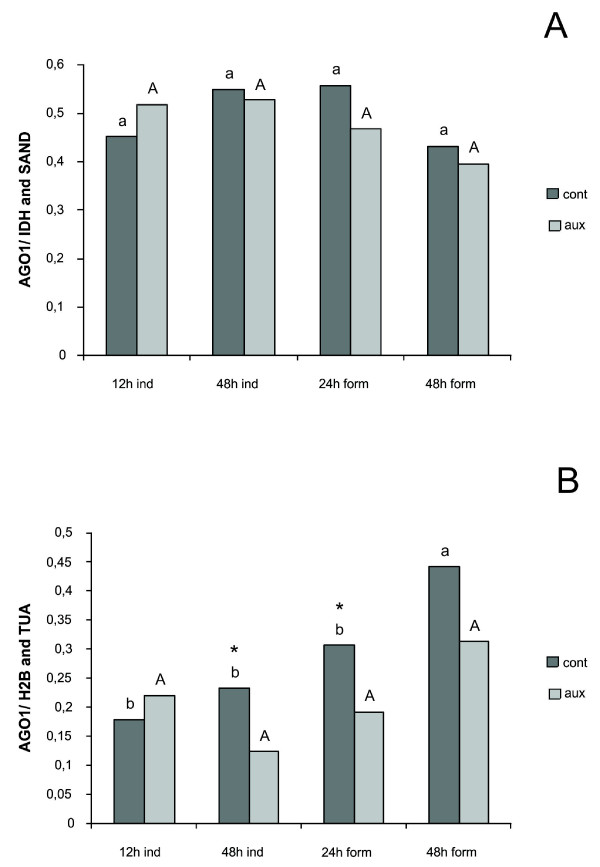
***AGO1 *relative expression profile during *Eucalyptus globulus in vitro *adventitious rooting**. The *AGO1 *(*Argonaute 1*) expression profile was investigated relative to the best combination of reference genes indicated by both *geNorm *(A) and *NormFinder *(B) programs. Aux: indicates addition of 10 mg l^-1 ^Indol Butyric Acid (IBA) in induction step culture medium; cont: indicates absence of IBA in induction step culture medium; Columns sharing the same letter are not different by ANOVA followed by Duncan test (*P *≤ 0.05); small letters correspond to analysis of variance (ANOVA) performed for control treatment samples; capital letters correspond to analysis of variance (ANOVA) performed for samples treated with auxin. *: indicates significant difference between treatments within time points by *t-test *(*P *≤ 0.05).

Using *IDH *and *SAND *as references (indicated by *geNorm*) *AGO1 *relative expression profile was not significantly different among the adventitious rooting time points analysed both in presence or absence of auxin (Figure 5A). When *H2B *and *TUA *were used as references (indicated by *NormFinder*), *AGO1 *relative expression at 48 h after transfer to formation step (48 h form) was significantly higher in absence of auxin (control), whereas no differences were detected for the auxin exposure treatment (Figure 5B). Moreover, when comparing presence and absence of auxin within each time point, *AGO1 *relative expression after 48 h of culture on root induction medium (48 h ind) and at 24 h after transfer to root formation medium (24 h form) was higher in absence of auxin (control) than in presence of auxin (Figure 5B). Thus, the control gene combinations suggested by each program yielded different relative expression profiles of a gene of interest.

## Discussion

In gene expression studies, qPCR is often the method of choice for a target gene expression profile investigation because it is currently one of the most sensitive techniques available [15-17,20]. In this context, a correct normalization is a pre-requisite for the reliability of the final results [59,60], especially considering possible biological relevance of small differences in gene expression or in the study of different tissues and organ samples [18].

In the present work, candidate reference genes for expression studies on adventitious rooting in *E. globulus *microcuttings were selected based on previous reports of normalization in plants, mainly with *A. thaliana*, *Populus *sp. and *Eucalyptus *sp., taking into account the model plant status of the first species and the woody habit of the last two genera. Additional genes frequently used for normalization in qPCR studies in other plant species were also evaluated.

The use of a single reference gene has been avoided to minimize the chances of erroneous expression estimates [24,57]. In fact, the use of multiple reference genes is becoming the golden standard in expression studies [19]. In order to select the most suitable reference genes for use in adventitious rooting, we investigated the relative expression profile of a gene of interest (*AGO1*) during this process, using the pairs of reference genes indicated as most stable by each one of the programs (*geNorm *and *NormFinder*).

An increase in *AGO1 *expression would be expected during microcutting root development considering its function in regulating auxin metabolism-related genes during adventitious rooting in *Arabidopsis *[10]. The higher *AGO1 *expression in the absence of auxin may be due to a lower requirement for the regulation of auxin-metabolism related genes in presence of exogenous auxin.

Therefore, *NormFinder *seemed to be better suited for the selection of the most stable reference genes for monitoring gene expression during adventitious rooting, detecting subtle differences that can be regarded as important for the adventitious rooting process. Recent studies have reported some discrepancies between different approaches for selection of reference genes and have chosen *NormFinder *[43,45,61,62] as data analysis tool. This is mainly due to the wide applicability of this tool in any kind of experimental design, especially when involving different cell types and experimental stages associated with high variation of gene expression [45,51]. Hence, we suggest *H2B *and *TUA *as the most adequate reference genes for expression studies during the rooting of *E. globulus *microcuttings.

*H2B *(Histone H2B) is one of the four core histones that form the mononucleosome, the fundamental repeating unit of chromatin in eukaryotic cells [63], being a key component for genetic material structure [52]. Two works have motivated us to include *H2B *in our analysis. Hays et al. [64] employed it as reference in Northern blot analysis to assess differential gene expression between alfalfa (*Medicago sativa *L.) varieties exhibiting long- or short-stalked glandular hairs. Later, Sterky et al. [65], through a transcriptomics approach, showed that transcripts for histones (and ribosomal proteins) were among the most ubiquitous gene products present in different tissues and species of *Populus*. More recently, a histone H3 gene was included when defining reference genes for the analysis of gene expression in longan tree somatic embryogenesis by qPCR [45]. Nevertheless, histone genes have been employed as references mostly for expression studies in animal systems [66,67].

The high expression stability showed in our experiments for *TUA *corroborates data of Brunner *et al. *[24] in a study with poplar. On the other hand, Tong and collaborators found *TUA *as one of the most variable genes for studies with peach, except when comparing genotypes or different storage time of fruit [40].

Data to date point to *IDH *as the most used control gene for normalization in *Eucalyptus *sp. [53-55], but without indication of a previous reference-gene detailed analysis. Although elected as the most stably expressed gene in *Eucalyptus *clones under biotic and abiotic stress [50], results from the present study did not support ranking *IDH *as one of the best reference genes because, together with SAND, it appears less sensitive to detect slight differences in the expression profile of an adventitious rooting-related gene of interest. This fact reinforces the need of detailed reference gene analysis for specific experimental conditions and processes.

Adventitious rooting is a very complex process, regulated by both environmental and endogenous factors, but the molecular mechanisms by which adventitious root formation is regulated are still poorly understood. Important advances are upcoming with genetic and microarray studies in *Populus*, especially after the availability of the poplar genome [68]. Studying gene expression during rooting in *Pinus contorta*, Brinker *et al. *[69] reported an increased expression of genes involved in protein synthesis and a decreased expression of genes related to protein degradation for the first 3 days after auxin treatment, whereas an opposite trend was observed during root formation and elongation. Sorin *et al. *also identified proteins with altered expression during adventitious rooting in *Arabidopsis *mutants, and, among these, there were some related to protein degradation [70]. These observations may help explain the low expression stability of the gene for polyubiquitin (*UBI*) found in our experiments (Figure 3 and Table 3), since the product of this gene is known to function in protein degradation [71].

The least stably expressed gene was *18S*, both by *geNorm *and *NormFinder *analysis (Figure 3 and Table 3). The use of rRNA as reference gene was previously considered a good strategy in gene expression studies [21] but recent works suggest that this is often not the case [24,72,29,40,44,45]. This is possibly due to the high abundance of *18S rRNA *compared with target mRNA transcripts, making it difficult to accurately subtract the baseline value in qPCR data analysis [24]. Besides, *18S rRNA *can not be used for this purpose when reverse transcription is carried out using oligo-dT primers or only mRNA is used as template [29].

The most stably expressed genes identified in this study, *H2B and TUA*, were effective references in both phases of the rooting process, induction and formation, and also in presence or absence of auxin, i.e. with conditions leading to profuse and scarce root development, respectively (Figure 2B and Figure 5B). This is a rather important feature because it allows the use of these genes as internal controls in evaluating the expression of putative key genes in the rooting process that are likely to vary between phases and in conditions leading or not to root development [73]. These key genes may include those encoding auxin transporters, auxin metabolism enzymes, auxin receptors and selective proteolysis-related proteins [74].

## Conclusions

This work constitutes the first in-depth study to validate optimal control genes for the quantification of transcript levels in *E. globulus *during *in vitro *adventitious rooting.

Considering that both programs used are based on distinct statistical algorithms, potential discrepancies could be expected. The comparative *AGO1 *expression profile, using the combinations of reference genes indicated by each program, allowed us to suggest the most adequate combination of control genes, based on the functions described in the literature for this gene of interest. Hence, the *NormFinder *program was considered more appropriate for our experiments, indicating *H2B *and *TUA *as suitable reference genes for normalization in *E. globulus *microcuttings rooted *in vitro*. Besides, *UBI *and *18S *are not indicated for use as internal controls in this species under the conditions tested.

In summary, these findings represent useful tools for normalization of qPCR results and will enable more accurate and reliable gene expression studies related to the vital process of clonal propagation by rooting in this important woody species.

## Methods

### Plant material

Seeds of *Eucalyptus globulus *Labill (batches from Chile and kindly supplied by Celulose Riograndense S.A., Guaíba, RS, Brazil) were surface-sterilized in 70% (v/v) ethanol (1 min) and 1.5% (v/v) NaClO (20 min) followed by 4 washes in sterile distilled water. Fifteen seeds were planted in 300 ml glass flasks (capped with a double layer of aluminum foil) containing 60 ml of medium as follows: half-strength modified MS salts [75] with the concentration of calcium chloride reduced to 1/6, 2% (w/v) sucrose, pH adjusted to 5.8 ± 1 and 0.6% (w/v) agar (extra pure microbiological grade, *Merck*) [8]. The medium was sterilized in autoclave for 20 min at 121°C. The growth room conditions were 16 h photoperiod, 30 μmol m^-2^s^-1 ^of photosynthetically active radiation (provided by white light fluorescent tubes) and temperature of 23 ± 2°C. After 14 weeks, tip microcuttings (about 3 cm in length) were excised from the seedlings and used for *in vitro *rooting experiments.

### *In vitro *adventitious rooting experiments

The culture system consisted of a two-step protocol: an initial step of induction, which lasted 96 h (induction medium composition: 0.3x MS salt concentration, 0.4 mg l^-1 ^thiamine HCl, 100 mg l^-1 ^inositol, zero (control) or 10 mg l^-1 ^indolyl-butyric acid (leading to rooting), equivalent to 49.3 μM, 30 g l^-1 ^sucrose and 6 g l^-1 ^agar, adjusted to pH 5.8 ± 1 before autoclaving), followed by a formation step (same composition of induction medium except without added auxin and supplemented with 1 g l^-1 ^activated charcoal) [8].

Experiments were carried out in 20 ml glass vials containing 6 ml of medium, which were capped with a double layer of aluminum foil, at a density of two explants per vial. Both treatments (presence and absence of auxin during the induction step) were carried out in a growth room with the same conditions described above for seed germination.

The expression analysis of the selected genes was monitored along the rooting process and the harvest of microcuttings for RNA extraction was at 12, 24, 48 and 96 h of exposure to induction medium and 24 and 48 h after transfer to formation medium (formation step), for both treatments (with and without auxin in the first step). For the formation step harvest, the microcuttings remained for 96 h in the induction medium before transfer to formation medium (see Figure 1 for details). The microcuttings were immediately frozen in liquid nitrogen and stored at -80°C until RNA extraction. Each point of harvest combined six microcuttings (approximately 200 mg of homogenized tissue fresh weight). The experimental design was completely randomized and the experiments were independently repeated three times with similar results.

### Total RNA extraction and first strand cDNA synthesis

Total RNA was isolated using NucleoSpin RNA Plant Kit (*Macherey-Nagel*) including DNAse I treatment, following the manufacturer recommendations, with modifications as follows: 200 mg of homogenized tissue fresh weight from each sample was divided in two fractions of 100 mg. Each fraction of 100 mg had its RNA extracted separately. After the extraction procedure, the corresponding fractions were joined again for RNA quantification. Total RNA concentration was determined using Quant-iT™ RNA Assay Kit and the Qubit fluorometer (*Invitrogen*) and quality was monitored by electrophoresis in 1% agarose gel (data not show). One independent cDNA synthesis was performed for all of the samples starting from 100 ng total RNA. First strand cDNA synthesis was performed using oligo-dT primers and reverse transcriptase M-MLV (*Invitrogen*) in a final volume of 20 μl. The final cDNA products were diluted 10-fold in RNAse-free distilled water prior to use in qPCR.

### PCR primer design

The sequences used to design the primer pairs, except *IDH*, whose primers were obtained from Carvalho *et al. *[55], represent putative orthologs in eucalypt of genes chosen for this study and are described in Table 1. For that, sequences placed in the *Arabidopsis thaliana *Database (http://www.arabidopsis.org) were subjected to a tBlastx against the *Genolyptus Project *Database (http://www.lge.ibi.unicamp.br/eucalyptus/) and the sequence with highest homology was selected. For all genes, primer pairs were designed using Oligo Perfect™ Designer software (*Invitrogen*) and are described in Table 2. The specifications were: melting temperatures (Tm) of 58 - 62°C, primer lengths of 20 nucleotides, guanine-cytosine contents of 45-55% and PCR amplicon lengths of 100-150 base pairs.

### qPCR conditions

The qPCR analyses were performed in fast optical 48-well reaction plates 0.1 ml (*MicroAmp™- Applied Biosystems*) using a StepOne™ Real-Time PCR System (*Applied Biosystems*) according to the manufacturer instructions. All of the reactions were carried out in quadruplicates for each cDNA sample and contained 10 μl 10-fold diluted cDNA template, 4.25 μl sterile water, 2 μl 10× PCR Buffer (*Invitrogen*), 1.2 μl of 25 mM MgCl_2 _(*Invitrogen*), 0.1 μl of 10 mM dNTP (*Invitrogen*), 2 μl SYBR Green (1:10,000, Molecular Probes, *Applied Biosystems*), 0.2 μl of each of the 10 μM forward and reverse gene-specific primers and 0.05 μl Platinum^® ^Taq DNA polymerase (5 U/μl, *Invitrogen*) in a final volume of 20 μl. This experiment was repeated three times in independent runs for all selected genes per time sample.

Reactions were incubated at 95°C for 5 min to activate the Platinum^® ^Taq DNA polymerase (*Invitrogen*), followed by 40 cycles of 95°C for 15 sec, 60°C for 10 sec, and 72°C for 15 sec. The specificity of the PCR was confirmed with a heat dissociation curve (or melting curve) from 60°C to 90°C, following the final PCR cycle. This procedure allowed us to verify that the resulting fluorescence originated from a single PCR product and did not represent primer dimerization formed during PCR or a non-specific product.

### Determination of reference gene expression stability

Two publicly available software tools, *geNorm *v. 3.5 [24] and *NormFinder *[51] were used to evaluate gene expression stability. Both tools require the transformation of Cq (quantification cycle) values to linear scale expression quantities. The Cq values were converted into relative quantities by the delta-Cq method [56] using the sample with the lowest Cq as calibrator and taking into account the amplification efficiencies for each primer pair obtained with *LinReg PCR software *[76]. The measures were then exported to Microsoft Excel.

### *geNorm *approach

The *geNorm *software is a Visual Basic Application (VBA) tool for Microsoft Excel and relies on the principle that the expression ratio of two ideal reference genes should be constant throughout the different experimental conditions or cell types [24]. This program estimates an expression stability value (*M*) for each gene, defined as the average pairwise variation of a certain gene with all other control genes in a given panel of cDNA samples. Genes with the lowest *M *values have the most stable expression and an ideal *M*-value must be preferably <0.5 [57,27]. Reference genes are ranked by *geNorm *through the elimination of the worst-scoring candidate control gene (the one with the highest *M *value) and recalculating of new *M *values for the remaining genes. At the end of this procedure, two candidate genes are always top-ranked because expression ratios are required for gene-stability measurements [24]. The *geNorm *program also allows the establishment of minimal number of control genes required for calculating an accurate normalization factor, as the geometric mean of their relative quantities. A pairwise variation of 0.15 is accepted as *cut-off *[24] below which the inclusion of an additional control gene is not required for reliable normalization.

### *NormFinder *approach

The *NormFinder *software [51], another VBA applet, uses a model-based approach for identifying the optimal normalization gene(s) among a set of candidates. This strategy is based on a mathematical model of gene expression that enables estimation of the intra- and inter-group variations, which are combined into a stability value. Candidate control genes with the minimal intra-group variation will have the lowest stability value and will be top ranked [51]. For adequate application of the *NormFinder *program, the sample sets were subdivided in two groups (presence and absence of auxin in the induction step of adventitious rooting) with each of them containing 6 samples.

### Determination of *AGO1 *expression profile

The putative *A. thaliana AGO1 *ortholog in *E. globulus *had its expression profile investigated in eight samples among the twelve analyzed in this study (12 and 24 h after inoculation in induction medium, and 24 and 48 h after transfer to formation medium, both in presence and absence of auxin in induction medium). The experiment was carried out with three biological replicates and the qPCR method and primer design strategy followed the same parameters used for the analysis of reference genes. The relative expression profile analysis was obtained through the delta-Cq method [56]. Analysis of Variance (ANOVA), followed by Duncan's test when appropriate (*P *≤ 0.05), were applied for samples of the same treatment (presence or absence of auxin). To compare the same time point of different treatments, a *t-test *was used (*P *≤ 0.05).

## List of abbreviations

AGO1: Argonaute 1; AUX1: Auxin Resistant 1; PIN: *Arabidopsis thaliana *PIN-Formed proteins; qPCR: reverse-transcription followed by quantitative real-time Polymerase Chain Reaction; RNA: Ribonucleic Acid; PCR: Polymerase Chain Reaction; Cq: Quantification cycle; Tm: melting temperature; S.D: Standard Deviation; VBA: Visual Basic Application; NF: Normalization Factor; NaClO: Sodium Hypochloride; cDNA: complementary Deoxyribonucleic Acid.

## Authors' contributions

MRA performed all the experimental procedures, data analysis and drafted the manuscript. CMR assisted on the execution of experimental procedures and data analysis. FKR and RAS helped designing primers and in data analysis. GP assisted in gene selection and sequence identification at the Genolyptus database. AGFN conceived and supervised the study and finalized the paper. All authors read and approved the final manuscript.
